# Early diagnosis of Parkinson’s disease: biomarker study

**DOI:** 10.3389/fnagi.2025.1495769

**Published:** 2025-05-09

**Authors:** Jing Li, Mei Zhang, Chuan-Qing Yu, Min Xue, Pan-Pan Hu

**Affiliations:** ^1^Department of Neurology, The First Hospital of Anhui University of Science and Technology (Huainan First People’s Hospital), Huainan, China; ^2^Department of Neurology, The First Affiliated Hospital of Anhui Medical University, Hefei, China

**Keywords:** Parkinson’s disease, biomarkers, blood, cerebrospinal fluid, saliva, urine, tears, imaging

## Abstract

Parkinson’s disease (PD) is a common chronic degenerative disease with age-dependent increasing prevalence in the elderly. Non-motor symptoms include sensory deficiencies, autonomic dysfunction, psychological and cognitive abnormalities; while motor symptoms are bradykinesia, myotonia, resting tremor, and postural balance difficulties. The clinical diagnosis of PD depends mainly on patients’ medical history and physical examination. It is highly important to realize early detection of PD, and biomarkers are a valuable tool in this regard. The present study reviewed the findings of researches from the last few years, involving the advancements in the study of PD biomarkers in blood, cerebrospinal fluid, saliva, urine, tears, imaging, and pathology.

## 1 Introduction

**Figure 1:**
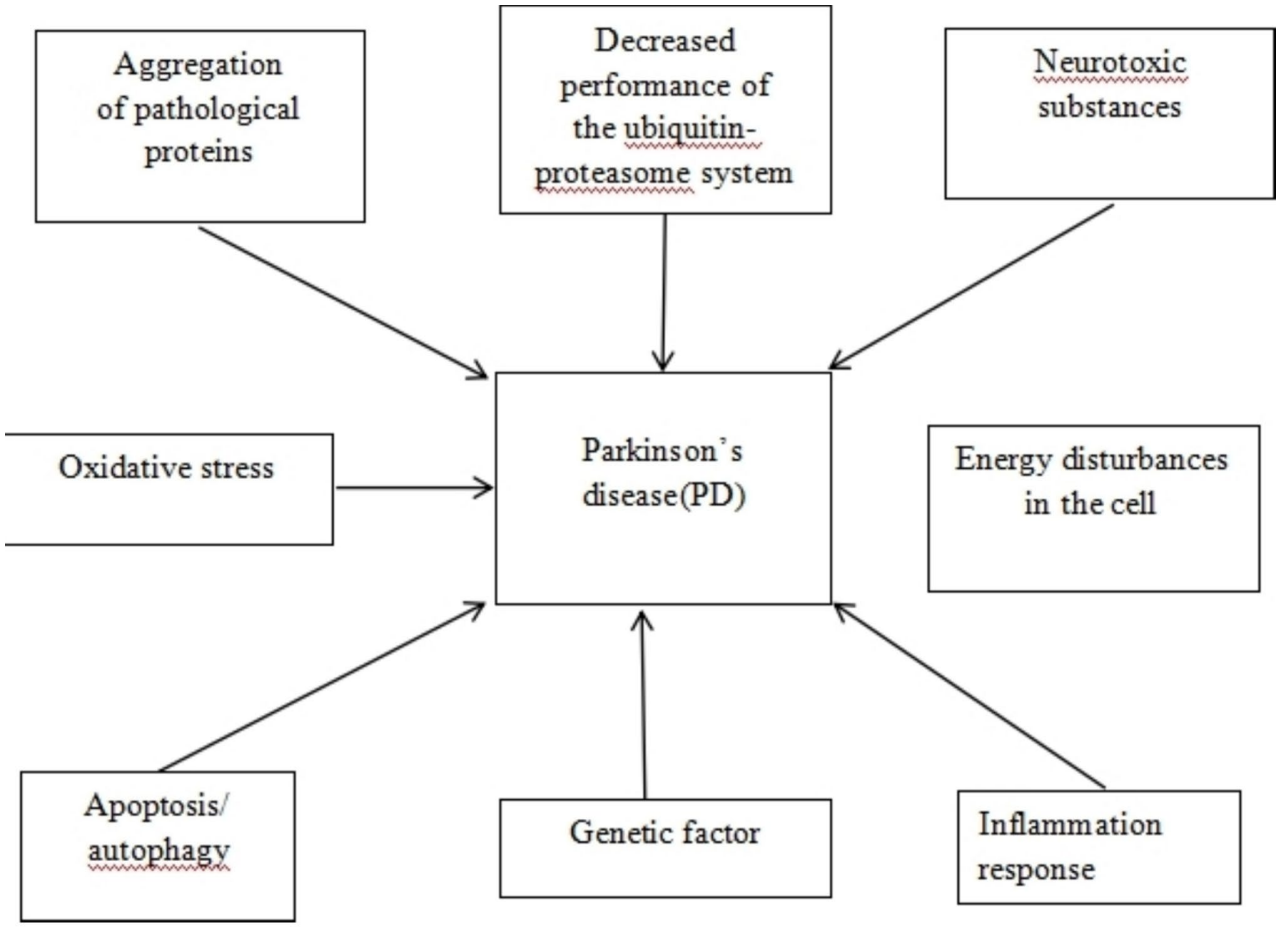
Mechanisms of neurodegeneration in Parkinson’s disease.

Parkinson’s disease (PD) has been accepted to be the second most common neurodegenerative disease worldwide after Alzheimer’s disease (AD), with Alpha-synuclein (*α*-Syn) deposition being its primary pathological manifestation. The primary pathophysiological mechanism of PD is dopamine dysfunction, leading to a series of motor and non-motor symptoms. So far, there is an absence of viable cure or way to halt its development. PD is one of the neurodegenerative illnesses with the greatest rate of growth, with an estimated global prevalence of 6 million individuals, which is expected to double by 2040 (Polissidis et al., 2020). Patients with PD may usually experience preclinical, prodromal, and clinically symptomatic stages. The diagnosis of PD is mostly based on core motor symptoms. However, PD has a prodromal phase that lasts for decades, resulting in a high possibility of early misdiagnosis (Tolosa et al., 2021). Patients are frequently identified with PD only in the presence of tremor and other symptoms, by which time 60–80% of the substantia nigra (SN) dopamine neurons have been lost, which occurs 5 to 15 years after the start of neuronal degeneration (Sulzer et al., 2018) (see Figure 1).

Given the current aging population, there will be a progressive rise in the number of PD sufferers in China. With the progression of PD to an advanced stage, PD patients may experience extremely poor he quality of life, causing a heavy financial burden on society. It necessitates the screening and identification of trustworthy and accurate PD markers to fulfill the goal of early diagnosis of PD. Biomarkers are biochemical indicators that can objectively measure and evaluate structural or functional changes in systems, organs, and cells. Useful indicators can mirror the occurrence, development, and prognosis of diseases during normal biological processes, pathogenic processes, or pharmacological responses to therapeutic interventions (Fereshtehnejad et al., 2017). As a result, more attention has been directed toward easily available biomarkers [blood, cerebrospinal fluid (CSF), saliva, tears, urine, imaging, pathology, etc.]. In this study, we describe the findings of recent domestic and foreign researches in terms of PD pathogenesis, biomarkers, and advances in the early detection of PD.

## 2 Blood biomarkers

### 2.1 Lewy body-related biomarkers

#### 2.1.1 α-Synuclein (α-Syn)

Eosinophilic inclusion bodies, or Lewy bodies, form in the cytoplasm of dopaminergic neurons remaining in the SN in patients with primary PD. α-Syn, a characteristic protein of Lewy bodies, is the earliest identified PD protein. α-Syn is a soluble protein that is usually expressed presynaptically in the central nervous system (CNS). The aggregation of α-Syn usually induces protein folding errors, which tends to aggregate into toxic oligomeric compounds, resulting in neurodegeneration in PD patients (Li P. Y. et al., 2021). Under oxidative stress, the aggregation of *α*-Syn monomers can result in α-Syn oligomers, further aggregating to form α-Syn fibers. These fibers eventually form Lewy bodies to induce the pathological manifestations of PD, thus disturbing SN, limbic system, olfactory bulb, etc. (Berg and Klein, 2023). Moreover, *α*-Syn is also subject to post-translational modification. Post-translational modifications to nucleoprotein, primarily phosphorylation, can result in phosphorylated *α*-Syn (p-α-Syn), thereby aggravating the toxicity and aggregation capacity of *α*-Syn (Choong and Mochizuki, 2022).

#### 2.1.2 Total α-Syn (t-α-Syn)

Controversial results remain regarding existing researches on plasma t-*α*-Syn in PD patients. For example, some researchers (Ding et al., 2017) proposed that PD patients had significantly higher plasma t-*α*-Syn levels than those of healthy controls; subsequent subgroup analyses also revealed significantly higher plasma α-Syn levels in PD patients with walking difficulties and postural abnormalities than those with tremor (Ding et al., 2017). Malek et al. (2014) documented no statistically significant difference in plasma *α*-Syn between PD patients and normal people, yet with a tendency of lower levels in PD patients. Moreover, patients with PD were reported to have lower peripheral blood levels of t-α-Syn, which was inversely linked to their motor impairments (Daniele et al., 2018). The *α*-Syn concentration in the control group was much lower than that of the PD group, whereas it was significantly higher in the patients with multiple system atrophy (MSA) (Dutta et al., 2021), revealing a potential in differentiating between PD and MSA, with a sensitivity of 89.8% and a specificity of 86.0%. In peripheral blood *α*-Syn stimulated cells, PD patients were detected with more IL-1β and IL-6 and less IL-10 than healthy controls, indicating an imbalance in the pro-inflammatory and anti-inflammatory cytokines in PD-associated neuroinflammation (Piancone et al., 2021). In a Bio-find cohort consisting of 46 PD patients and 45 healthy controls, a composite biomarker, consisting of t-*α*-Syn, anti-proteinase K (PKres) α-Syn, and p-α-Syn (PSer 129), was a strong indicator of differentiating PD patients from controls, with an area under the curve (AUC) of 0.81. Another study also supported *α*-Syn as a potential PD biomarker (Elhadi et al., 2019). The AUC of Ser129 p-*α*-Syn oligomer/total p-α-Syn was 0.69, with a sensitivity of 60.0% and a specificity of 59.5%. The AUC of α-Syn oligomer/t-α-Syn in blood exosomes was 0.71 for diagnosing PD (Zheng et al., 2021). However, the drawn conclusions are not universally agreed upon, possibly because *α*-Syn is primarily present in platelets and erythrocytes, and contamination or hemolysis can misrepresent the results.

#### 2.1.3 The α-Syn phosphorylation

Modifications of *α*-Syn at the post-translational level (e.g., phosphorylation, ubiquitination, and nitration) can enhance its aggregation ability. Under normal physiological conditions, there may be relatively low phosphorylation level of the Ser129 site of *α*-Syn; however, in the pathological state of PD, the Ser129 site is almost entirely in the phosphorylation modification state. Generally, 90% of phosphorylated *α*-Syn is deposited in Lewy bodies, and only 4% of α-Syn is phosphorylated in normal brain tissue. Therefore, the p-α-Syn may be an important diagnostic biomarker for PD (Ramalingam and Dettmer, 2023). A study measured serum p-Ser129-*α*-Syn by recruiting 10 patients with early PD and 11 healthy controls. The AUC of p-Ser129-α-Syn in diluted human serum was 0.92, suggesting its potential value as a biomarker for clinical diagnosis of early PD (Chen et al., 2022). Ser129-α-Syn (pS-α-Syn) levels in the erythrocytes of PD patients were significantly higher than those of healthy controls, with an AUC of 0.96, and a relatively high sensitivity (93.39%) and specificity (93.11%). Further subgroup analysis revealed significantly higher pS-*α*-Syn levels in patients with late-onset PD than those with early-onset disease, as well as in patients with postural instability and gait difficulties than those with tremor-predominant PD, but obviously lower in patients with cognitive deficits and olfactory loss with PD (Li X. Y. et al., 2021). In addition, another histological analysis was performed in rats 6 months later after the injection of *α*-Syn protofibers into the medial forebrain bundle. It was observed with a significant reduction in the amount of dopamine transporter proteins in the striatum, as well as a decrease in the volume of the right cortical, bilateral limbic, and right hippocampal regions in the experimental group of rats, while the thalamus showed an increase. Therefore, damage to the limbic system and thalamic fiber structures may occur in the early stages of PD (Wang Y. et al., 2020).

#### 2.1.4 Oligomeric α-Syn

Oligomeric α-Syn, originating from aberrant aggregate, exhibits a wide range of neurotoxicity and can operate through non-cell-autonomous routes involving glial cells that are either chronically or dysfunctionally activated. *α*-Syn oligomers cause massive degeneration and loss of nigrostriatal dopaminergic neurons, in turn inducing PD through multiple mechanisms, involving mitochondrial dysfunction, endoplasmic reticulum stress, proteasomal influences, neuroglial cellular and inflammatory responses, membrane damage, synaptic dysfunction, autophagy and lysosomal dysfunction (Bengoa-Vergniory et al., 2017). PD Patients had higher levels of phosphorylated tau and oligomeric *α*-Syn in their erythrocytes (Daniele et al., 2018). With an AUC of 0.76, a sensitivity of 79.0% and a specificity of 64.7%, the ratio of α-Syn oligomer to total RBC protein was found to be considerably higher in patients with PD compared to the controls (Wang X. M. et al., 2015). In addition, in relative to the controls, patients with early or even prodromal PD had considerably greater levels of t-α-Syn, haploid α-Syn, and oligomeric α-Syn in their blood (Katunina et al., 2023).

### 2.2 Oxidative stress-related biomarkers

#### 2.2.1 Protein deglycase-1 (DJ-1)

The PARK7 gene encodes a multifunctional protein known as DJ-1, whose structure and function can be changed by mutations in PARK7, resulting in an autosomal recessive illness that raises the risk of PD. DJ-1 plays roles an antioxidant and oxidative stress sensor, contributing to mitochondrial homeostasis; moreover, it also regulates several signaling pathways, transcription factors, molecular chaperone functions, apoptosis, autophagy, and dopamine homeostasis (Skou et al., 2024). Patients with untreated PD were detected with higher blood oxidized DJ-1 protein (oxDJ-1) levels. Meanwhile, the PD group had the highest levels, followed by the progressive supranuclear palsy (PSP) group and then the MSA group, compared with the control group (Yamagishi et al., 2018). In patients with early PD, higher levels of oxidized DJ-1 were found in erythrocytes and dopaminergic neuronal cells in the midbrain SN (Saito and Noguchi, 2016). Blood levels of 4-hydroxy-2-nonenal (4-HNE)-modified DJ-1 were significantly higher in PD patients than those in controls, which were even higher in patients with advanced PD than those in patients with early PD. Therefore, this indicator may also be associated with disease severity, as evidenced by different levels of DJ-1 in blood from PD patients at different stages and healthy controls (Lin et al., 2012).

#### 2.2.2 Uric acid (UA)

UA is one of the primary antioxidants in the human body, which is the byproduct of purine metabolism and has the ability to scavenge oxygen radicals, chelate metal ions, and lower oxidative stress level. Patients with PD had significantly lower serum UA levels (Kodama and Katoh, 2017). Animal experiment also documented correlations of serum UA levels with lower striatal dopamine levels, tyrosine hydroxylase-positive neuronal death in the SN pars compacta, and the degree of oxidative stress in the midbrain. A decreased risk of PD development was also reported to be linked to higher serum UA levels (Sarukhani et al., 2018). Serum UA levels were considerably lower in patients at risk of developing PD (UA: 261.6 μM, controls: 338.7 μM) according to a search for blood alterations during the prodromal period of PD (Katunina et al., 2023). Another cross-sectional study using an electronic medical record database found that lower serum UA levels increased the incidence of PD, sped up the disease course, and aggravated motor problems and cognitive impairment with long-term dopaminergic medication (Dănău et al., 2022). Serum UA levels also decreased with worse sleep quality in PD patients, suggesting its significance as a biomarker of sleep disturbance in PD patients (Zhou et al., 2023). In addition, a meta-analysis of 13 studies involving 2,379 PD patients and 2,267 controls revealed the relationship of UAwith PD, low blood UA levels were more likely to cause PD, and patients with early-stage PD had significantly higher serum UA levels than those with intermediate- and late-stage PD patients, which is thought to be an indirect predictor for the development of PD (Wen et al., 2017).

#### 2.2.3 8-hydroxy-2′-deoxyguano-sine (8-OHdG)

Reactive oxygen radicals attack the carbon atom at the 8-position of the guanine base in the DNA molecule, producing the oxidation product 8-OHdG. This mechanism provides an essential evidence for interpreting the aging processes, degenerative disorders, and other connections with oxidative stress. The formation of reactive oxygen species (ROS) has been recognized to be a contributor to PD, and oxidative stress is a major part in the pathophysiology of the disease. It also participate in the degradation of dopaminergic neurons. According to a prior study, PD patients were observed with much greater levels of 8-OHdG in peripheral blood leukocytes than those of the healthy controls, and this difference was connected with the severity of PD (Chen et al., 2009). Studies of PD patients with varying levels of cognitive function revealed that blood 8-OHdG levels were significantly higher in both PD non-demented and demented patients than those in normal controls (Bogdanov et al., 2008; Stoessel et al., 2018). Another clinical study revealed higher blood 8-OHdG levels in unmedicated PD patients than those of the controls. Thus, measurement of blood 8-OHdG levels in PD patients exhibits possible importance for researching the connection between oxidative stress and PD.

#### 2.2.4 Coenzyme Q10 (co Q10)

Co Q10 is an antioxidant that is soluble in fat, and an electron acceptor for complex I of the mitochondrial respiratory chain enzyme. Co Q10 is regarded as an important antioxidant owing to its trophic and protective effects on dopamine neurons. The equilibrium relationship between Co Q10 and its oxidized forms is upset under pathological circumstances. Both *in vitro* and *in vivo* studies have revealed that abnormalities in the mitochondrial complex may disrupt the redox equilibrium, and that a reduction in Co Q10 concentration may increase free radicals without scavenging them, thereby inducing neuronal degeneration (Mischley et al., 2016). The changes in CoQ10 may be related to the damage to the blood–brain barrier (BBB). In a rat model of PD, CoQ10 treatment improved the motor function of rats, raised the number of TH + cells in the striatum, and slowed down the damage to dopaminergic neurons (Ghasemloo et al., 2021). As a result, Co Q10 considerably slowed down the progression of PD, which can be considered as a biomarker in clinical settings, and the diagnosis of PD may benefit from the determination of decreased Co Q10 activity. Additionally, the levels of CoQ10 were shown to be significantly lower in PD patients when compared to controls, suggesting that CoQ10 may be a possible biomarker of antioxidant status (Mischley et al., 2012). However, in another trial, in contrast to controls who took an oral placebo, PD patients who took Co Q10 orally for a while did not exhibit a statistically significant improvement in their condition (Beal et al., 2014), which deserves our further exploration and verification.

### 2.3 Inflammatory immune response-related biomarkers

#### 2.3.1 Interleukin (IL)

IL is a lymphokine that can stimulate and control immune cell proliferation and differentiation, which is a significant contributor to the inflammatory response. From the perspective of pathophysiology, PD shows obvious inflammatory response. Among 44 PD patients and 22 healthy people, Scalzo et al. (2010) compared and discovered significantly higher IL-6 levels in PD patients than those in controls, and that the PD patients with relatively higher IL-6 levels had a slower stride and were more prone to fatigue. In a PD model, the modeled rats were observed with compromised BBB and markedly elevated IL-17A levels in the SN (Li et al., 2019). In a study involving 230 people with PD who had just started the disease and 93 healthy controls, subjects were assessed on scales at baseline, 18 months, and 36 months, respectively, based on the measurement of cytokines. TNF-*α*, IL1-*β*, IL-2, and IL-10 were significantly higher in the blood of PD patients than in the control group, suggesting the predictive value of these biomarkers in predicting the course of PD (Williams-Gray et al., 2016). Another study collected the blood samples of 45 patients with early-stage PD and 20 healthy controls to measure IL-1β, IL-2, IL-6, IL-10, and TNF-α levels, with significantly lower levels of IL-2 and IL-6 in the control group than those in PD patients (Kim et al., 2022). Patients with PD had considerably higher blood levels of TNF-α and IL-6 than the control group. Serum IL-6 levels were linked to depression, cognitive dysfunction, and age of onset, while serum TNF-α levels would predict the stage of disease and age of onset (Fu et al., 2023). In addition, patients with PD had higher serum levels of hs-CRP, IL-1β, and IL-6 when compared to controls (Zhang et al., 2023).

#### 2.3.2 C-reactive protein (CRP)

CRP is a microprotein mostly produced in the liver, recognizing as a nonspecific indicator of the acute phase of the inflammatory response. Tissue injury raises serum CRP levels, which are mostly controlled by IL-6. CRP was determined to be a pivotal biomarker for determining the onset and severity of PD (Mehta et al., 2023). In addition to being significantly higher in PD patients who had frozen gait, prior research also established an association of ultrasensitive CRP with non-motor symptom and H&Y staging (Santos-García et al., 2019). Patients with PD had noticeably greater blood levels of hypersensitive CRP than those of normal controls (Baran et al., 2018). In a cross-sectional study of 204 patients with PD and 204 healthy controls, blood levels of hypersensitive CRP were significantly higher in patients with PD than those in controls (Yang et al., 2020). Another case–control study also documented a close correlation of blood CRP levels with motor function scores (Sawada et al., 2014).

#### 2.3.3 Homocysteine (Hcy)

In the process of methylation, there will be natural production of the amino acid HCY, which is controlled by folate levels and a range of hereditary variables. It also stimulates astrocytes and microglials, resulting in neuroinflammation. The HCY level was monitored to be risen as PD developed and progressed (Fan et al., 2020). Hcy levels were considerably higher in PD patients with cognitive loss in a case–control study involving 246 individuals (Periñán et al., 2022). Men with PD or AD were documented, in a meta-analysis, to have higher levels of Hcy than women (Nguyen et al., 2023). Considering the association of elevated HCY levels with PD risk of PD, supplementation of vitamin B6 and B12 is recommended for patients on a long-term levodopa/dopa decarboxylase inhibitor (DDI) regimen (Phokaewvarangkul et al., 2023). Higher levels of Hcy were found to be significantly and positively linked with cognitive dysfunction in PD patients (Fenoy et al., 2016). PD hallucinations was also reported to connect elevated HCY levels, especially in men (Zhong et al., 2022).

#### 2.3.4 Neutrophil-to-lymphocyte ratio (NLR)

NLR has been believed to be a possible biomarker of inflammatory state. NLR was considerably greater in PD patients than controls (Muñoz-Delgado et al., 2021). Lower dopamine transporters in the caudate nucleus and nucleus accumbens were shown to be linked with higher NLR in 211 confirmed PD and 344 new-onset PD (Muñoz-Delgado et al., 2023). Besides a higher NLR in PD patients than controls, NLR was found to be positively connected with disease course and inversely correlated with the amount of amyloid-β42 in the CSF (Grillo et al., 2023). In a case–control study involving 303 PD patients and 303 healthy controls, NLR was significantly higher in PD patients than in controls. Additionally, NLR showed a positive correlation with the Unified PD Rating Scale (UPDRS I, II, III) and Hoehn Yahr staging, representing a negative correlation with the MMSE and MoCA (Li et al., 2024). In addition, a meta- analysis of retrospective and prospective trials, involving both Caucasians and Asians, confirmed higher NLR in PD patients than in controls (Hosseini et al., 2023).

### 2.4 Neurotrophic factor-related biomarkers

Neurotrophic factors function to regulate microglia, regenerate axon, stimulate remyelination, and nourish neurons. Neurotrophic factors are classified into multiple families, including motor neuron neurotrophic factors, glial-derived nerve growth factor family, neurotrophin family, and non-specific neurological classes of trophic factors. The emergence of gene therapy and neurotrophic factor augmentation therapy may benefit PD treatment.

#### 2.4.1 Brain-derived neurotrophic factor (BDNF)

BDNF is the most abundant in hippocampus and cortical regions of the CNS. Serving as a neuroprotective and neuromodulatory molecule, BDNF gives dopaminergic neurons trophic assistance. Inhibited BDNF production may diminish patients’ cognitive function and alter nigrostriatal dopaminergic neuron structure. BDNF differed between PD and normal populations, which was lower in PD patients than in normal populations, also exhibiting an association with depressive symptoms (Wang Y. et al., 2017). A meta-analysis of 1,034 cases of PD and 886 healthy controls showed that BDNF levels were significantly lower in PD patients than controls (Chen and Zhang, 2022). Additional data also documented an association between BDNF and cognitive decline in PD patients, and it was hypothesized that a reduction in BDNF was associated with cognitive decline in PD patients (Wang Y. et al., 2022).

#### 2.4.2 Insulin-like growth factor-1 (IGF-1)

Insulin also regulates cell growth, similar to IGF-1 and IGF-2. IGF-1 is mostly produced by the liver and delivered into the bloodstream, Its physiological function is generally controlled by IGF-1 receptor and IGF-binding proteins. By improving neuronal metabolism and controlling neuronal excitability, IGF-1 can protect dopaminergic neurons from potential damage. Patients with early PD were shown to have higher IGF-1 levels, which were linked to cognitive impairment, anxiety, and depression (Shi et al., 2022). Moreover, PD patients had higher serum IGF-1 expression, with a significant correlation between IGF-1 levels and cognitive function scores as well (Ma et al., 2015). Another study also revealed a noticeably higher serum IGF-1 levels in PD patients than those of normal controls (Castilla-Cortázar et al., 2020). Collectively, IGF-1 can be a useful biomarker for determining PD risk in humans.

#### 2.4.3 Epidermal growth factor (EGF)

With 53 amino acid residues, EGF is a single-chain, low-molecular polypeptide that is heat-resistant. EGF binds to the particular EGF receptor on those cells, ultimately encouraging target cell mitosis and DNA synthesis. Plasma EGF levels fall in the early stages of PD, but rise later in the disease’s progression. Furthermore, plasma EGF levels correlated with early motor and non-motor symptoms of PD patients, and were considerably higher in PD patients than in those of idiopathic tremor patients (Jiang et al., 2015). Lower plasma EGF levels were linked to higher likelihood of cognitive deterioration at follow-up. Moreover, a substantial correlation was discovered between plasma EGF levels and cognitive functioning in a study involving 236 PD cases and 396 AD cases (Lim et al., 2016).

### 2.5 Genetic markers

Monitoring of genetic markers is still an effective approach to screen the population at risk of PD and early stage patients, even if single gene mutations account for only a small percentage of cases (5–10%). Possible pathogenic variants include autosomal dominant (SNCA, and LRRK2) and autosomal recessive (Parkin, PINK1, and DJ-1) mutations. Gene products at the transcriptional and post-transcriptional levels, in addition to the mutation itself, can reveal the underlying pathophysiological processes of PD (Tönges et al., 2022). In cases of familial late-onset PD, 8.67% exhibited mutations in GBA (heterozygous), HTRA2, and SNCA, while 30.03% had disease-causing genes in CHCHD2, DJ-1, GBA (heterozygous), LRRK2, PINK1, and PRKN (Sun et al., 2023).

The expression of SNCA, one gene related to PD, is specific to neurons and blood cells. Locascio et al. (2015) documented in their study, with the involvement of 500 PD patients and 363 healthy people, that reduced SNCA transcript was linked to cognitive loss in PD patients during follow-up. Lower SNCA transcripts might be a possible predictor of cognitive decline, and low SNCA transcripts were linked to early PD. Furthermore, microRNAs (miRNAs) have also been identified as possible biomarkers of PD as they can pass across the BBB. Downregulation of miRNA19b was detectable up to 4.5 years prior to a final PD diagnosis (Fernández-Santiago et al., 2015), suggesting its potential as an early diagnostic tool for PD. Alieva et al. (2015) compared peripheral blood mRNA expression in patients with early-stage PD to healthy subjects. As a result, patients with PD had higher levels of ATP13A2 antibody, recombinant human PD protein 7, and zinc-finger protein ZNF746 in their peripheral blood than did controls, suggesting that these elevated mRNAs might serve as early PD indicators. Further research has demonstrated the differential role of *α*-syn in patients with early PD from healthy individuals, and that a persistent decline in the transcript level of SNCA can signify the advancement of PD. Therefore, these biomarkers can both offer diagnostic value for an early PD and track the disease’s course (see Table 1).

**Table 1:** Candidate biomarkers in blood (plasma or serum) of patients with PD.

PD mechanism	Biomarker	Compared to healthy subjects	Indicative of the course of the disease	References
Louis Minor (name)	Total alpha-syn	↑	Associated with postural abnormalities, gait difficulties	Ding et al. (2017), Malek et al. (2014), Daniele et al. (2018), Dutta et al. (2021), Piancone et al. (2021), Elhadi et al. (2019), Zheng et al. (2021)
	Phosphorylated alpha-syn	↑	Associated with disease progression, postural abnormalities, gait difficulties	Ramalingam and Dettmer (2023), Chen et al. (2022), Li X. Y. et al. (2021), Wang et al. (2020)
	Oligomeric α-syn	↑	Correlates with disease progression	Bengoa-Vergniory et al. (2017), Wang X. M. et al. (2015), Katunina et al. (2023)
Oxidative stress	DJ-1	↑	Correlates with disease severity	Skou et al. (2024), Yamagishi et al. (2018), Saito and Noguchi (2016), Lin et al. (2012)
	UA	↓	Associated with disease progression, cognitive function, sleep disorders	Kodama and Katoh (2017), Sarukhani et al. (2018), Dănău et al. (2022), Zhou et al. (2023), Wen et al. (2017)
	8-OHdG	↑	Correlates with disease severity	Chen et al. (2009), Bogdanov et al. (2008), Stoessel et al. (2018)
	Co Q10	↓	Correlates with degree of illness	Mischley et al. (2016), Ghasemloo et al. (2021), Mischley et al. (2012), Beal et al. (2014)
Inflammatory immunity	IL-6	↑	Presented with slowed gait, easy fatigue, depression, cognitive decline, associated with motor and non-motor function scores	Scalzo et al. (2010), Williams-Gray et al. (2016), Fu et al. (2023)
	TNF-α	↑	Associated with age of onset and H&Y staging	Fu et al. (2023)
	IL-1β, IL-2, IL-10,	↑	Associated with disease progression	Williams-Gray et al. (2016)
	CRP	↑	Correlates with Freezing Gait, Hallucinations, Motor Function, and Non-Motor Symptom Scores	Mehta et al. (2023), Santos-García et al. (2019), Baran et al. (2018), Yang et al. (2020), Sawada et al. (2014)
	HCY	↑	Related to cognitive function, hallucinations	Fan et al. (2020), Periñán et al. (2022), Nguyen et al. (2023), Phokaewvarangkul et al. (2023), Fenoy et al. (2016), Zhong et al. (2022)
	NLR	↑	Correlates with disease duration, H&Y staging, motor function, cognitive function	Muñoz-Delgado et al. (2021), Muñoz-Delgado et al. (2023), Grillo et al. (2023), Li et al. (2024), Hosseini et al. (2023)
Neurotrophic factor	BDNF	↓	Related to cognitive function	Wang Y. et al. (2017), Chen and Zhang (2022), Wang Y. et al. (2022)
	IGF-1	↑	Associated with anxiety, depression and cognitive impairment	Shi et al. (2022), Ma et al. (2015), Castilla-Cortázar et al. (2020)
	EGF	↑	Related to cognitive function	Jiang et al. (2015), Lim et al. (2016)
Genetics	GBA	↑	Associated with risk of developing PD	Tönges et al. (2022), Sun et al. (2023)
	SNCA	↓	Sustained lowering predicts disease progression	Locascio et al. (2015)
	microRNA	Down-regulation of mRNA19b	Monitoring of disease severity	Fernández-Santiago et al. (2015), Alieva et al. (2015)
		ATP13A2, PARK and ZNF746 upregulation		

## 3 Cerebrospinal fluid (CSF) markers

### 3.1 Biomarkers related to Lewy bodies

#### 3.1.1 α-Synuclein (α-Syn)

CSF is a clear bodily fluid that can most closely mimic brain function. However, it is a challenge to determine α-Syn in CSF, depending on careful sample collection and processing. The measurement of α-Syn may be compromised greatly by lumbar puncture technique, collection tube material manufacturer, storage environment, and time. Neuropathologically speaking, PD is a gradual death of dopaminergic neurons in the dense region of the SN, typically accompanied by the formation of Lewy synapses and intra-neuronal Lewy bodies. *α*-Syn is the primary protein found in Lewy bodies and Lewy synapses, which has been recognized to be the most significant protein in the pathophysiology of PD (Tofaris, 2022). An evaluation in 78 PD patients and 32 controls showed considerably lower CSF t-*α*-Syn levels in PD patients (Førland et al., 2020). Chahine et al. (2020) found similar outcomes in another comparison between 21 healthy controls and 59 PD patients. This result can be explained partially by the SN’s α-Syn deposition and aggregation. The fibrotic aggregation of α-Syn in brain tissue may lower the quantity of total α-Syn that is available to enter the humoral circulation, resulting in a drop of α-Syn level in the CSF (Mollenhauer et al., 2019). The concentration of α-Syn in the CSF of PD patients was remarkably lower than that of normal participants, with the diagnostic sensitivity and specificity of 53.5 and 76.8%, respectively (Shi et al., 2014). Meanwhile, p-α-Syn at the Ser129 site (pS129α-Syn) and α-Syn oligomers (oligo α-Syn) were also extensively explored in PD, apart from the t-α-Syn. Patients with PD had higher levels of oligo α-Syn (o-α-Syn) and o-α-Syn/t-α-Syn ratio in CSF (Majbour et al., 2016a). Higher levels of post-translationally modified p-α-Syn (p-αS) were also detected in the CSF of PD patients than that of controls (Majbour et al., 2016b). Many post-translationally altered forms of α-Syn, such as SUMO-modified α-Syn, glycosylated α-Syn, nitrated α-Syn, and Tyr125-p-α-Syn, have the potential to be exploited for early PD diagnosis (AUC 0.84) (Miranda et al., 2017). In addition, protein misfolding cyclic amplification in conjunction with real-time shock-induced conversion analysis was used to identify aggregated and misfolded *α*-Syn in CSF. This technique demonstrated a high level of diagnostic accuracy (AUC 0.93 for PMCA and 0.89 for RT-QuIC) (Kang et al., 2019), along with an extremely high sensitivity (95.3%) and specificity (98%) (Rossi et al., 2020).

#### 3.1.2 *β*-amyloid (Aβ) and tau proteins

Aβ accumulation in multiple brain regions and hyperphosphorylation of tau proteins are also pathological alterations in PD, resulting in neuroprogenitor fibril tangles (Wills et al., 2010). In PD patients, tau and Aβ proteins can interact with *α*-Syn to enhance each other’s aggregation and deposition, hastening the course of cognitive impairment (Knopman and Can, 2022). The 42 amino acids that make up Aβ42 are primarily degraded amyloid precursor protein, which can be aberrantly deposited outside of neuronal cells. PD patients were detected with lower levels of Aβ42 in their CSF, which could also indicate severe cognitive impairment from PD (Irwin et al., 2020). Moreover, Aβ42 is a consistently measurable biomarker, supporting the significance of tracking Aβ in CSF in predicting cognitive decline in the early stages of PD. In addition, despite the decreasing trend in PD patients, CSF Aβ42 is still higher than that in patients with PD dementia (PDD), AD, or dementia with Lewy bodies (DLB). Therefore, this indicator may still be regarded as a useful tool for differentiating between patients with PD and those with dementia-like disorders (Dos et al., 2018).

Tau proteins attach to microtubule proteins to facilitate the production of microtubules, in turn forming the neuronal cytoskeleton. Neuronal fibrous tangles are created when Tau proteins are phosphorylated abnormally. Total tau (t-tau) and phosphorylated tau (p-tau) are two major tau proteins detected in the CSF. A prior cross-sectional investigation revealed lower t-tau and p-tau levels in CSF (Mollenhauer et al., 2017). The primary reason may be that tau protein is less concentrated in the free state after p-tau forms neurogenic fiber tangles. A two-year follow-up research comprising 42 patients revealed the correlation of *α*-Syn with the development of motor symptoms and cognitive impairment. Moreover, p-tau proteins was reported to suggest a worsening of motor symptoms, while Aβ was linked to declines in cognition, particularly memory (Hall et al., 2015). Additional evidence also unveil that elevated tau levels were associated with deteriorating motor symptoms and cognitive decline, primarily in the form of executive function impairment (Fereshtehnejad et al., 2023). Despite limited value of individual tau protein biomarkers in the early diagnosis of PD, the t-tau/t-α-Syn ratio, when combined with t-α-Syn levels in CSF, can greatly increase the accuracy of early PD diagnosis by allowing for better differentiation from AD or DLB (Førland et al., 2020).

#### 3.1.3 Ubiquitin carboxy-terminal hydrolase L1 (UCH-L1)

UCH-L1 is a component of the ubiquitin-proteasome system. Aberrant UCH-L1 protein expression has been detected in patients with familial PD. The UCH-L1 level in CSF of PD patients was considerably lower when compared to normal persons. Moreover, UCH-L1 could be utilized to distinguish between individuals with PD and normal people, with the AUC of 0.89 (Mondello et al., 2014). Moreover, there was a positive correlation between α-Syn and UCH-L1 levels in the cerebral fluid of PD patients (Wang K. et al., 2017). UCH-L1 combined with α-Syn can be accepted as a potential PD biomarker.

#### 3.1.4 Human kallikrein 6 (hK6)

Human kallikrein 6 is a CNS member of the serine protease family. It is one of the enzymes for cleavage-modification of α-Syn, widely distributed in brain tissue and CSF, and mostly found in the form of 25 ku zymogen (Kumar and Lee, 2023). The amount of hK6 was observed to be reduced in the brain of α-Syn transgenic mice (Spencer et al., 2013). Similarly, PD and AD patients had lower-than-normal hK6 content in the SN region (Ogawa et al., 2000). Besides, there was a substantial correlation between hK6 levels and α-Syn content in the CSF of patients with α-Syn-related diseases, including PD, DLB, and PDD (Wennström et al., 2013).

#### 3.1.5 Neurofilament light chain (NFL)

NF, made up of three subunits (light, medium, and heavy chains), is a structural protein that is essential for nerve impulse conduction, and maintains the integrity of neuronal morphology. It can also be employed as a biomarker for axonal degeneration brought on by acute neural tissue injury and neurodegenerative disorders (Graham et al., 2021). Axonal injury in the CNS might raise the level of NfL in the CSF (Kawarabayashi et al., 2023), hence, from a pathophysiological perspective, the degree of axonal injury or degeneration was positively connected with the amount of NfL in CSF. While other atypical Parkinson’s syndromes (APS), such as PSP, multisystemic atrophy (MSA), and corticobasal ganglion syndrome (CBS), are characterized by a marked increase in NfL level of CSF due to myelin axonal involvement, this pathological process is not typical in the early stages of PD (von Widekind et al., 2020). In a study of CSF NfL levels in a longitudinal multicenter cohort, patients with PSP and MSA had higher levels of CSF NfL than the PD group, whereas the control group had lower levels of NfL (Mollenhauer et al., 2020). Another systematic review of 15 studies confirmed the high diagnostic accuracy of CSF NfL levels in separating PD from APS 100. Therefore, CSF NfL can be a valid biomarker for differentiating between PD and APS.

An accurate assessment of the amount of elevated NfL in CSF may be hampered by the fact that CSF NfL may also increase with age due to degenerative changes in axons, even though its elevated levels correspond with the severity of cognitive impairment in patients (Lerche et al., 2020; Aamodt et al., 2021). In the future, it may be considered by lowering the upper age limit for study subjects to be included in, or performing a large prospective cohort study of NfL levels in normal CSF to create a baseline of fluctuating NfL levels that can be used as a control in subsequent research.

### 3.2 Neurotransmitter-related biomarkers

In general, patients with PD have degeneration of dopaminergic neurons in the SN compacta, decreased dopamine levels in striatal areas, and decreased levels of the dopamine metabolites homovanillic acid (HVA) and dihydroxyphenylacetic acid (DOPAC). Therefore and theoretically, a straightforward and practical PD diagnosis can be made based on neurotransmitter levels However, dopamine is compensatorily transported to terminals through other pathways during dopaminergic neuron injury; moreover, CSF dopamine levels are below conventional detection limits. As a result, CSF dopamine levels do not accurately indicate CNS dopamine deficiency Simultaneously, although changes in HVA in the striatum are not fully consistent with dopamine levels, DOPAC is a major metabolite of dopamine, suggesting that DOPAC in the CSF is more suitable for use as a diagnostic indicator of PD than dopamine and HVA (Landau et al., 2022). In the past, both DOPAC and HVA levels in CSF were reported to be significantly lower in PD patients compared to normal subjects, and correlated with motor deficits. Besides, lower levels of DOPAC were found in the CSF from patients with sporadic PD who had a positive striatal PET (18F-DOPA) scan. Accordingly, DOPAC in CSF is predicted to be a PD biomarker and can sensitively distinguish between PD patients (Kremer et al., 2021).

### 3.3 Lysosomal enzyme biomarkers

The two primary degradation mechanisms in the human body are the lysosomal and proteasome systems. Three primary forms of autophagy, macroautophagy, microautophagy, and molecular chaperone-mediated autophagy, are mediated by lysosomes, which are important organelles for the breakdown of proteins (Mizushima and Levine, 2020). The most frequent genetic risk factor for PD is mutations in the GBA gene, which codes for the enzyme lysosomal acidic *β*-glucoserebrosidase (G Case). Other lysosomal storage disorder-associated genes are also linked to PD. Therefore, lysosomal metabolic markers can be promising biomarkers for PD. In PD patients, there was a significant reduction in CSF G Case, histone D, and β-hexokinase activities, regardless of the presence or absence of GBA mutations. The sensitivity and specificity of histone D were 61 and 77%, and those of G Case were 67 and 77%, respectively (Parnetti et al., 2017). Early detection of PD was possible, with 82% sensitivity and 71% specificity, when using G Case activity, o-*α*-Syn/t-α-Syn, and age jointly (Parnetti et al., 2014). As a result, possible lysosomal biomarkers may be involved when combining biomarkers covering several pathologic pathways of PD for diagnosis.

### 3.4 Oxidative stress-related biomarkers

#### 3.4.1 Protein deglycase-1 (DJ-1)

DJ-1 plays a role in mitochondrial dysfunction in PD. A study found that patients with PD had lower levels of DJ-1 than both normal subjects and AD patients, with the generated sensitivity and specificity of 90 and 70% for the diagnosis of PD, respectively (Zhao et al., 2010). Moreover, extensive Lewy bodies in the SN and bluestriata neurons were observed in the postmortem examination of an early- onset PD patient with low levodopa efficacy. A genetic investigation of the PARK7 gene also revealed a considerably decreased amount of DJ-1 due to the p.L172Q mutation (Taipa et al., 2016). In addition, according to Herbert et al. (2014), lower levels of DJ-1 in CSF was examined in individuals with PD than patients with multisystemic atrophy. The difference between the two groups could be distinguished with an AUC of 0.84, sensitivity of 78%, and specificity of 78%.

#### 3.4.2 Uric acid (UA)

Elevated UA might increase DNA expression and cellular activity in animal models of PD *in vivo* and *in vitro*, suppressing cell division and encouraging neurogenesis. UA played multiple functions in the onset and progression of PD (Lee J. E. et al., 2022). When treating PD patients with thalamus substrate nucleus deep brain stimulation (STN-DBS), UA-associated functional brain connectivity can be employed to evaluate the therapeutic outcome (Chang et al., 2023). Allantoin is one of the oxidized products of UA. UA levels in serum and CSF were lower in individuals with new-onset PD, but allantoin and allantoin/UA ratios were higher in these patients than in controls, both of which were correlated with Rapid eye movement sleep behavior disorder (RBD) and autonomic function (Hasíková et al., 2023). Furthermore, the overall correlation coefficient between UA and Aβ42 was 0.67 in the CSF of normal individuals, while it was 0.49 in the CSF of patients with non-demented PD. As a result, there might be positive correlation between CSF UA levels and Aβ42, which was thought to have a role in altered cognitive performance through the Aβ42-related pathway (Maetzler et al., 2011).

#### 3.4.3 8-hydroxy-2′-deoxyguano-sine (8-OHdG)

8-OHdG has been considered an accurate indicator of oxidative stress. Research has demonstrated higher 8-OHdG levels in CSF of non-demented PD patients than those in normal participants (Gmitterová et al., 2009). Subjects with mutations enriched for the leucine repeat kinase 2 (LRRK2) gene had higher quantities of 8-OHdG and 8-ISO in their cerebral fluid (Loeffler et al., 2017). Moreover, 8-OHdG concentrations were found to be much higher in the CSF of PD patients than in controls, which could further increase as the course of the disease prolonged (Isobe et al., 2010). The 8-OHdG concentrations in both the CSF and blood were significantly higher in PD patients than in the normal subjects, with higher concentrations in non-demented PD patients. Additionally, the 8-OHdG concentrations were also correlated with the course of PD (Gmitterová et al., 2018).

#### 3.4.4 Coenzyme Q10 (co Q10)

In the CSF of PD patients, the ratio of oxidized CoQ10 to total CoQ10 (%CoQ10) was much greater than in normal participants, and the size of the ratio corresponded with the course of the disease (Isobe et al., 2010). Furthermore, in mice at elevated risk of PD, continuous Co Q10 injection dramatically decreased cerebral levels of protein carbonyl, and improved animal performance on a number of behavioral tasks (Attia and Maklad, 2018). CoQ10 supplementation, however, produced none obvious enhancement in the motor function or quality of life in PD patients, according to a meta-analysis of 981 people (Negida et al., 2016).

### 3.5 Biomarkers of neuroinflammation

#### 3.5.1 Interleukin (IL)

Microglia (MGC) area group of resident macrophages of the CNS that are susceptible to pathophysiologic damage. MGCs get activated in case of morphological and phenotypic alterations brought on by injury. Activation of inflammatory vesicles in microglia by *α*-synucleinogen fibers occurs early in the development of PD, leading to an inflammatory cascade and MGC proliferation. To scavenge pathogenic pathogens, these MGCs may boost the release of inflammatory factors, chemokines, ROS, and nitric oxide. In addition, severe or protracted MGC activation may cause dopaminergic cell death, hastening the onset of PD (Magnusen et al., 2021; Lee S. et al., 2022). PD patients were reported to have higher CSF TNF-α levels (Schröder et al., 2018; Majbour et al., 2020). Besides, significantly greater CSF levels of transforming growth factor β1 (TGF-β1), IL-1β, and IL-6 were also observed in PD patients than those in controls, according to a systematic study of CSF cytokines in PD patients (Chen et al., 2018).

#### 3.5.2 C-reactive protein (CRP)

The degree of raised CRP levels in CSF has been reported to be strongly correlated with the severity of non-motor symptoms in PD patients. Moreover, significantly higher CRP levels were documented in patients with dementia from PD and multisystemic atrophy than those in PD and control groups. This finding further supports that elevated CRP levels are suggestive of cognitive impairment progression in PD (Hall et al., 2018). Furthermore, a phenotypic analysis of immune cell profiles, using multiparametric flow cytometry, revealed higher proportions of activated T lymphocytes and non-classical monocytes (CD14 +/CD16 +) in the CSF of PD patients than controls (Schröder et al., 2018).

#### 3.5.3 YKL-40

In the CNS, YKL-40 is a glycoprotein produced in microglia and astrocytes, which is discovered to modulate inflammatory responses. According to a prospective research, it rose as the illness worsened and was linked to patients’ cognitive deterioration (Llorens et al., 2017). Its results in PD, however, remain controversial. In an animal investigation, elevated levels of YKL-40 were examined in the cerebral fluid of rats with PD (Anwar, 2023). Cytomics study revealed that PD patients had greater amounts of YKL-40 in their CSF compared to normal persons (Gevezova et al., 2023). Additionally, YKL-40 levels were found to be lower in PD patients than those of controls (Hall et al., 2018).

#### 3.5.4 Monocyte chemoattractant protein 1 (MCP-1)

Monocyte chemoattractant protein 1 is a chemokine that promotes the recruitment of monocytes and the spread of inflammation. In a cohort study comprising 46 PD patients, 17 MSA patients, and 52 healthy controls, the severity of the disease increased with the amount of MCP-1 in the CSF of PD patients (Santaella et al., 2020).

### 3.6 Metabolomics and proteomics biomarkers

Small molecule metabolite remains the center in the study of metabolomics, and modifications in metabolite levels may be the final result of controlling protein expression. PD-specific metabolic fingerprints have been found by CSF metabolite analysis, all of which may be used as PD diagnostic indicators to certain extent. So far, the documented primary early PD metabolic pathway alterations include oxidative stress (significantly lower levels of dehydroascorbic acid than controls), carbohydrate metabolism (significantly higher levels of fructose, mannose, and threonine), lipid metabolism (e.g., sphingolipids or phospholipids), amino acid metabolism (lower levels of 3-hydroxyisovaleric acid and tryptophan and higher levels of leucine, isoleucine, and ketoleucine than controls), and fatty acid oxidation (lower levels of creatinine) (Wang J. et al., 2022; Stoessel et al., 2018; Trezzi et al., 2017; Trupp et al., 2014; Wuolikainen et al., 2016; Willkommen et al., 2018). Nevertheless, clinically significant biomarkers have not been revealed in non-targeted metabolomics, and the primary issue is the large heterogeneity in metabolites amongst studies. Considering that abnormal protein aggregation is one of the main hallmarks of PD, non-targeted proteomics may offer valuable data to comprehensively identify the PD. Similar to metabolomics, there is a great deal of inter-study variation in positive proteins, necessitating further research to determine their clinical importance (see Table 2).

**Table 2:** Candidate biomarkers for cerebrospinal fluid in patients with PD.

PD mechanism	Biomarker	Compared to healthy subjects	Diagnostic value	References
Louis Minor (name)	Total alpha-syn	↓	Related to motor symptoms	Førland et al. (2020), Chahine et al. (2020), Mollenhauer et al. (2019), Shi et al. (2014)
	Oligomeric α-syn	↑	Correlates with severity of illness	Majbour et al. (2016a)
	Phosphorylated alpha-syn	↑	Correlates with severity of illness	Majbour et al. (2016b)
	Aβ_42_	↓	Associated with cognitive impairment	Knopman and Can (2022), Irwin et al. (2020), Dos et al. (2018)
	t-tau, p-tau	↓	Associated with exacerbation of motor symptoms	Mollenhauer et al. (2017), Hall et al. (2015), Fereshtehnejad et al. (2023)
	UCH-L1	↓	Positively correlated with α-Syn	Mondello et al. (2014), Wang K. et al. (2017)
	hK6	↓	Positively correlated with α-Syn	Spencer et al. (2013), Ogawa et al. (2000), Wennström et al. (2013)
	NF-L	↑	Differentiating PD from atypical Parkinson’s syndrome in relation to cognitive functioning	Kawarabayashi et al. (2023), von Widekind et al. (2020); Mollenhauer et al. (2020), Angelopoulou et al. (2021), Lerche et al. (2020), Aamodt et al. (2021)
Neurotransmitter	DOPAC	↓	Related to movement disorders	Landau et al. (2022), Kremer et al. (2021)
Lysosomal enzyme	G Case	↓	/	Parnetti et al. (2017), Parnetti et al. (2014)
Oxidative stress	DJ-1	↓	Lower than AD and MSA	Zhao et al. (2010), Taipa et al. (2016), Herbert et al. (2014)
	UA	-	Positively correlated with Aβ42 and associated with disease progression	Lee J. E. et al. (2022), Chang et al. (2023), Hasíková et al. (2023), Maetzler et al. (2011)
	8-OHdG	↑	Correlates with disease duration	Gmitterová et al. (2009), Loeffler et al. (2017), Isobe et al. (2010), Gmitterová et al. (2018)
	%CoQ10	↑	Correlates with disease duration	Attia and Maklad (2018), Negida et al. (2016)
Neuroinflammation	IL-1β, TGF-β1, IL-6, TNF-α	↑	-	Schröder et al. (2018), Majbour et al. (2020), Chen et al. (2018)
	CRP	↑	Related to cognitive function	Lindqvist et al. (2013), Hall et al. (2018), Schröder et al. (2018)
	YKL-40	↑	Related to cognitive function	Llorens et al. (2017), Anwar (2023), Gevezova et al. (2023)
	MCP-1	↑	Correlates with disease progression	Santaella et al. (2020)
Metabolomics and proteomics	3-hydroxyisovaleric acid, tryptophan levels	↓	The metabolites varied from study to study and the clinical significance needs to be further explored.	Wang J. et al. (2022), Stoessel et al. (2018), Trezzi et al. (2017), Trupp et al. (2014), Wuolikainen et al. (2016), Willkommen et al. (2018)
	Leucine, Isoleucine and Ketoleucine	↑		
	Dehydroascorbic acid	↓		
	Fructose, mannose and sulforaphane	↑		
	creatinine	↓		

## 4 Saliva markers

### 4.1 *α*-Synuclein (α-Syn)

Different from common monomeric form in healthy persons, α-Syn is partially expressed as oligomers in PD patients. Lewy bodies occur as a result of these oligomers developing into adult amyloid fibers (Wilson et al., 2019). PD patients had significantly decreased salivary α-Syn concentrations overall compared to healthy controls. Furthermore, significant correlations of the length, stage, and severity of the disease were documented with t-α-Syn concentration in PD patients, suggesting that the concentration of this biomarker could be a predictor of the disease’s progression (Vivacqua et al., 2016; Shaheen et al., 2020; Figura and Friedman, 2020). A study quantified the concentrations of several types of α-Syn, such as t-α-Syn, oligomeric α-Syn, and phosphorylated ser129 α-Syn, in the salivary exosomes of individuals with PD. It was discovered that PD patients had higher levels of oligomeric α-Syn and a higher ratio of oligomeric α-Syn to t-α-Syn in the saliva than those of controls (Cao et al., 2019). Moreover, significantly higher levels of oligomeric *α*-Syn and oligomeric α-Syn/t-α-Syn were also reported in the saliva of PD patients. All these data imply that salivary oligomeric α-Syn concentrations may also serve as a potential biomarker for monitoring the course of PD in individuals (Kang et al., 2016).

### 4.2 Protein deglycase-1 (DJ-1)

A mutation in the 189-amino acid protein DJ-1 was reported to be related to a rare family autosomal recessive PD (Farah et al., 2018). Under normal conditions, DJ-1 is mostly located in the cytoplasm of dopaminergic neurons, with lower protein levels detected in the nuclei and mitochondria. In response to oxidative stress, DJ-1 monomers polymerize to create dimers, aiding in the protein’s localization in mitochondria and migration to the nucleus ultimately. Under mild-to-moderate oxidative stress in cells, DJ-1 recruitment at the plasma membrane and mitochondria was thought to be the sole way to protect neurons effectively (Maita et al., 2013). A study enrolling 285PD patients and 91 healthy controls to measure DJ-1 concentrations in the saliva using Luminex. The results showed an elevation in the levels of this protein in PD patients, as well as a substantial correlation between salivary DJ-1 and H&Y staging. Furthermore, compared to individuals with tremor and motor, people with mixed PD had a much lower concentration of DJ-1 in their saliva (Kang et al., 2014). Another study also measured the salivary DJ-1 concentrations in PD patients using western blotting. The results showed a significant rise in this concentration in PD patients relative to healthy controls, and this increase was associated with the severity of the disease (Masters et al., 2015). However, in other study detecting the salivary concentration of DJ-1 using ELISA, higher levels were found in PD patients than that in the control group, yet without statistically significant difference (Devic et al., 2011). Collectively, salivary DJ-1 levels may not be a reliable indicator of the presence of PD in healthy persons. Taking into consideration of inconsistent experimental results currently, further research is needed to ascertain whether DJ-1 may be used as a salivary biomarker for the diagnosis of PD.

### 4.3 Heme oxygenase-1 (HO-1, also known as HSP32)

Increased HO-1 levels may cause an excessive accumulation of iron and carbon monoxide, resulting in oxidative stress and cellular damage. Such increase may also trigger the formation of *α*-Syn via dopaminergic neurons (Cressatti et al., 2019). In PD, HO-1 was measured to be increased in nigral astrocytes and in neuronal Lewy bodies (Schipper et al., 2019). In primary astrocyte cultures, glial HO-1 activation enhanced pathological iron accumulation and mitochondrial oxidative damage (Wang J. et al., 2015). Moreover, HO-1 may stand for some clinical characteristics of PD, including neuroinflammation, oxidative stress, dysregulated iron metabolism, and mitochondrial damage. In addition, salivary HO-1 concentrations in patients with early-stage PD appeared to be considerably greater than in healthy controls (Song et al., 2018). Therefore, salivary HO-1 concentrations might offer useful evidence to enable the distinguishing between patients with early-stage PD and healthy controls. All together, an elevated level of HO-1 in saliva may function as a biomarker for early identification of PD.

### 4.4 MiR-153 and miR-223

Non-invasive assays based on miRNA in patient saliva stand out regarding their diagnostic significance in PD or even specific subtypes of the illness. For example, a prior research constructed a transgenic mouse model overexpressing HO-1 presenting with clinical symptoms resembling those of PD patients in humans (Cressatti et al., 2020). In this model, miR-153 and miR-223, which were down-regulated in the brain and serum, controlled the expression of α-Syn. Salivary levels of miR-153 and miR-223 were determined by reverse transcriptase quantitative PCR (qRTPCR). PD patients had lower salivary levels of both miRNAs than those of the controls, suggesting their potential as disease-specific biomarkers. However, so far, there is still one study examined the salivary levels of miR-153 and miR-223, requiring further validation to confirm their noninvasive diagnostic value for PD.

### 4.5 Acetylcholinesterase activity (AChE)

Patients with concurrent symptoms of reduced salivation and dry mouth have been observed as a consequence of employing AChE as a diagnostic biomarker for PD. In an early exploratory study, a significant reduction in salivary flow rate and an increase in AChE activity were observed in PD patients compared to controls, with a clear positive correlation with H&Y staging. Unfortunately, the study only described the pathophysiological potential of parasympathetic denervation in PD patients, owing to the constraint of technical problems, rather than demonstrating that AChE activity testing gives an acceptable clinical diagnostic for PD diagnosis (Fedorova et al., 2015) (see Table 3).

**Table 3:** Saliva candidate biomarkers in PD patients.

Biomarker	Compared to healthy subjects	Indicative of the course of the disease	references
Total alpha-syn	↓	Correlates with disease duration, stage, and severity of disease	Vivacqua et al. (2016), Shaheen et al. (2020); Figura and Friedman (2020)
Oligomeric α-syn	↑	Correlates with disease progression	Cao et al. (2019), Kang et al. (2016)
DJ-1	↑ or no change	Correlates with disease severity and staging	Kang et al. (2014), Masters et al. (2015), Devic et al. (2011)
HO-1	↑	-	Song et al. (2018)
MiR-153 and mir-223	↓	-	Cressatti et al. (2020)
AChE	↑	Staging with H&Y	Fedorova et al. (2015)

## 5 Urine markers

### 5.1 Urine metabolomics markers

Upon examining clinical urine metabolites, PD patients exhibited significant irregularities in their metabolism of tryptophan, nucleotides, phenylalanine, steroidogenesis, fatty acid *β*-oxidation, histidine, and tyrosine. Furthermore, in a Drosophila model of PD, alterations in the tryptophan metabolic pathway were in line with the overexpression of *α*-Syn in the disease (Luan et al., 2015; Luan et al., 2014). The data from the previously described study illustrates the use of urine-based metabolomics technology for identifying metabolite characteristics and associated changes in metabolic pathways that characterize PD pathophysiology. The accompanying alterations associated with cross-species PD may offer addition evidence for understanding the molecular metabolic control of PD. Another research intended to identify difference in the metabolism of tyrosine, tryptophan, and glutamate through the detection of 20 compounds in the urine of PD patients and controls using ultra-high performance liquid chromatography–tandem mass spectrometry combined with *in situ* selective derivatization. The results revealed significant differences in the levels of ethanol dehydrogenase (ADH) and phenylethanolamine N-methyltransferase (PNMT) between groups (Lee et al., 2017). Moreover, succinic acid concentrations in the urine of PD patients were associated with motor symptoms. Furthermore, in 100 PD and 50 normal subject, significant increases were noticed in the concentrations of ornithine, phenylalanine, isoleucine, β-hydroxybutyric acid, tyrosine, and succinic acid detected by high-resolution nuclear magnetic resonance spectroscopy (Kumari et al., 2020). In addition, liquid chromatography-mass spectrometry was also used to test the urine of 104 PD patients and 111 controls. Compared to the controls, PD patients had higher contents of 3-methoxytyramine, N-acetyl-L-tyrosine, orotate, and urine vanillic acid xanthine, but lower levels of imidazolylactate 3,3-dimethylglutarate (Wang et al., 2023) (see Table 4)

**Table 4:** Urine biomarkers in PD patients.

Biomarker	Compared to healthy subjects	Indicative of the course of the disease	References
Phenylethanolamine N-methyltransferase (PNMT) and ethanol dehydrogenase (ADH)	↑	-	Lee et al. (2017)
Ornithine, phenylalanine, isoleucine, beta-hydroxybutyric acid, tyrosine and succinic acid	↑	Related to motor symptoms	Kumari et al. (2020)
3-methoxytyramine, N-acetyl-L-tyrosine, orotate, urovanillic acid xanthine	↑	-	Wang et al. (2023)
3,3-Dimethylglutaric acid imidazololactic acid	↓	-	Wang et al. (2023)

## 6 Tear markers

Tears, or lacrimal fluid, are a transparent, slightly milky, watery fluid released by the lacrimal gland and conjunctival cup cells. The main organic components of tears are proproteins, which include albumin and globulin, Lysozyme, immunoglobulin IgA, IgG, IgE, etc. In general, PD patients exhibit reduced facial expression, fewer fluttering eye movements, smaller tears, and different tear composition (Safranow et al., 2014). According to a previous analysis of tear fluid from 18 normal controls and 36 PD patients using mass spectrometry, PD patients had considerably greater amounts of 21 proteins and lower levels of 19 proteins than those of the normal subjects. Moreover, these proteins detected were found to be linked to immunological response, lipid metabolism, and oxidative stress in PD (Boerger et al., 2019). Another study analyzed tear fluid from 24 PD patients and 27 healthy persons, with the identification of 560 tear proteins using nano-liquid chromatography-mass spectrometry. Pro-vitamin A/C, histone enzyme D, acidic ceramidase, transitional endoplasmic reticulum ATPase, cytoplasmic dynamin 1, whose expression was elevated, and tripeptidyl peptidase 1 were among the lysosomal functions-related proteins discovered among these (Acera et al., 2022). Thus, biomarkers possibly associated with PD may be discovered by examining any alterations in protein production.

In another research, tears from 20 PD patients and 20 healthy controls were collected and analyzed by mass spectrometry immunoassay, with the discovery of higher amounts of p-tau in tears of PD patients (Molfetta et al., 2023). Upon analyzing the constituents of tears in PD patients as well as in a mouse model, tears exhibited markedly elevated norepinephrine and α-2-macroglobulin activity. Additionally, the limb on which motor symptoms initially appeared was positively correlated with the elevated levels of adrenaline (Bogdanov et al., 2021) (see Table 5).

**Table 5:** Tear biomarkers in patients with PD.

Biomarker	Compared to healthy subjects	Indicative of the course of the disease	References
Previtamin A/C (LMNA), histone D (CATD), acid ceramidase (ASAH1), transitional endoplasmic reticulum ATPase (TERA), cytoplasmic dynamin 1 (DYHC1)	↑	-	Acera et al. (2022)
Tripeptidyl peptidase 1 (TPP1)	↓	-	Acera et al. (2022)
p-tau	↑	Correlates with severity of illness	Molfetta et al. (2023)
Norepinephrine levels	↑	Correlates with location of motor symptoms	Bogdanov et al. (2021)
Alpha-2-macroglobulin activity levels	↑	Correlates with location of motor symptoms	Bogdanov et al. (2021)
Adrenaline level	↓	Correlates with location of motor symptoms	Bogdanov et al. (2021)

## 7 Imaging biomarkers

### 7.1 Imaging markers based on ultrasound

Transcranial ultrasonography, with a diagnostic sensitivity of 84% and specificity of 85%, can identify anomalies of nigral iron deposition in PD patients, which present as nigral hyperechoicity (Tao et al., 2019). But in 10–20% of PD patients, the temporal bone sound window was of poor quality, suggesting that transcranial ultrasonography may not be a superior choice for diagnosis in this population.

### 7.2 Imaging markers based on magnetic resonance techniques

Concerning PD, the principal magnetic resonance imaging markers are diffusion tensor imaging, free water imaging, and neuromelanin imaging in the SN and locus coeruleus. There are also additional imaging changes in PD patients, which, however, still necessitate further investigation to develop and disseminate sophisticated imaging and analytic methods, as well as to carry out multi-center, large-sample size studies to create novel imaging indicators, so as to validate their clinical relevance. The pathogenesis of PD is attributed primarily to the loss of dopaminergic neurons in the SN of midbrain. Nigrosome 1 has been found to be the first and most severe site of dopaminergic neuron loss in PD, which is found in the caudal mid-lateral part of the midbrain SNpc. Subsequently, the loss of neurons can spread to other SN pars compacta and other brain regions, including the ventral tegumental area, the locus coeruleus, the dorsal nucleus of the vagus nerve, and other neuromelanin (NM)-rich regions (Damier et al., 1999), of which NM is a ferric ion-protective chelating agent. The SN experiences an increase in free iron content and a drop in paramagnetic NM-iron complex level with neuronal death and NM depletion. It may further increase paramagnetism in magnetic susceptibility-weighted imaging (SWI). Therefore, early identification of PD can be realized by identifying the loss of NM and the rise in free iron by quantitative magnetic susceptibility maps (QSMs), SWI, etc. based on NM-MRI (magnetic resonance imaging) (Wypijewska et al., 2010). PD patients have been found with reduced total NM volume indicated by NM-MRI (Mueller et al., 2014; Pyatigorskaya et al., 2018). In another study on PD patients and healthy controls based on NM-MRI, there was significant reduction in the lateral, central, and medial appearance of the NM high-intensity signals in the SN in patients with early PD (Wang et al., 2018). The dorsolateral SN Nigrosome 1 hyperintense vanished in the QSM and SWI of PD patients, commonly referred to as the “swallow-tail sign” disappearance. The NM signal area was considerably smaller in patients with PD for 2–5 years compared to those with early PD (Fabbri et al., 2017). In addition, for PD diagnosis, NM-MRI had a mean specificity of 83% (95% CI 76–88%) and a sensitivity of 89% (95% CI 86–92%) (Cho et al., 2020).

Diffusion tensor imaging (DTI) provides details on the movement of water molecules at the neuronal level, which can facilitate the evaluation of the integrity and connectivity of white matter fibers. Early microstructural alterations could be determined based on variations in fractional anisotropy scores in the SN, striatum, and thalamus substrate nucleus vary in DTI images of PD patients (Pyatigorskaya et al., 2017). Compared to DTI, free-water imaging offers more accurate information on neuronal volume, axonal density, and degree of damage, and can examine free-water molecules in brain tissue. Moreover, elevated free water has been observed in the posterior portion of the SN in RBD patients, as well as in early-stage PD patients (Zhou et al., 2021; Ofori et al., 2015). In a multicenter PPMI cohort, free water in the posterior region of the SN increased in *de novo* PD from baseline to 1, 2, and 4 years, and changes in free water within 1 year predicted changes in the Hoehn & Yahr scale at 4 years (Burciu et al., 2017). Therefore, free-water imaging is anticipated to serve as an imaging marker for PD early diagnosis, RBD conversion prediction, and PD progression prediction.

### 7.3 Imaging markers based on molecular imaging

By tagging synapses, neurotransmitters, and glucose transporters at the molecular level, positron emission tomography (PET) and single photon emission computed tomography (SPECT) are common molecular imaging techniques to reflect cerebral functional changes. This type of imaging modalities is primarily used to identify potential dopaminergic dysfunction in populations at high risk of PD and to differentiate between PD and other Parkinson’s syndromes.

#### 7.3.1 Molecular imaging markers based on dopamine transport and metabolism-related molecules in presynaptic neurons of dopaminergic neurons

Dopaminergic transporters (DAT) are transporters of dopamine found on the presynaptic membrane of dopaminergic neurons. Therefore, visualization of DAT may be beneficial to measure dopamine deficit in PD. Striatal DAT deficiency is commonly assessed by DAT PET ligands like ^11^C-CFT, ^18^F-CFT, ^11^C-PE2I, and ^11^Caltropane, as well as SPECT ligands like ^123^I-Iodoflupan and ^123^I-*β*– CIT, ^123^I-FP-CIT, with 99–100% sensitivity and specificity in identifying dopamine deficits in the early stages of PD (Palermo and Ceravolo, 2019). These ligands are valuable tools for PD diagnosis in the clinical setting. The enzyme known as aromatic amino acid decarboxylase (AADC), whose visualizer is ^18^F-DOPA, is essential to dopaminergic neurons as it can catalyze the conversion of levodopa into dopamine. The Food and Drug Administration of the United States authorized^18^F-DOPA PET, with high sensitivity, in 2019 as a dependable diagnostic technique at advanced stages of PD (Dhawan et al., 2022; Kaasinen et al., 2019). Furthermore, the presynaptic membrane contains vesicular monoamine oxide transporter 2 (VMAT2), which is responsible for the reuptake of monoamine neurotransmitters (dopamine and 5-hydroxytryptamine) into presynaptic neurons. Its developer is ^18^F-FP-DTBZ. In addition to being utilized for condition monitoring, VMAT2 imaging can identify striatal dopaminergic depletion in individuals with early PD (Hsiao et al., 2014).

#### 7.3.2 Glucose transporter (GLUT)-based molecular imaging markers

Glucose transporter uses the visualizer^18^F-FDG PET to examine glucose metabolism in the brain, exhibiting higher accuracy than MRI at detecting anomalies in brain activity. The ^18^F-FDG PET pattern known as PD-related pattern is unique to PD patients. Its imaging features include a relative increase in metabolism in the pallido-thalamic, pontine, and cerebellar regions, along with a decrease in metabolism in the pre-motor and posterior parietal regions; and it has a high degree of sensitivity and specificity (93.9% for both) (Meles et al., 2019).

#### 7.3.3 Molecular imaging markers based on neuroinflammation

PD-associated visualizers also target neuroinflammation. The benzodiazepine receptor is expressed on the outer membrane of active microglia and macrophage mitochondria. It is a major regulator of microglial activity and a hallmark of inflammation in the nervous system. It is visualized by ^18^F-DPA-714 and ^18^F-GE-180. PD patients were examined with elevated signals in nigrostriatal, pontine, basal ganglia frontal, and temporal cortex (Viviano et al., 2022; Alam et al., 2017). However, PET targeting neuroinflammation just reveal inflammation and cannot be used to precisely diagnose early PD, requiring research to determine its clinical value.

#### 7.3.4 Molecular imaging markers based on 5-hydroxytryptaminergic and glutamatergic systems

Non-dopaminergic biomarkers may enable the detection of pathologic alterations associated with PD in stages 1–2, in case of involvement of dopaminergic neurons in PD, or Braak 3. PD depression, which typically manifests early in the disease, is linked to the serotonergic system. The serotonin transporter PET ligands ^11^C-DASB and ^11^C-WAY100635 are useful in identifying individuals at risk of PD prior to the presence of dopaminergic system degradation (Fu et al., 2022; Wilson et al., 2019). However, so far, it is still unclear whether this imaging technique can differentiate between PD and non-PD depression.

#### 7.3.5 Molecular imaging markers based on peripheral sympathetic nerve

Peripheral noradrenergic sympathetic nerves are the target of ^123^I- iodophenylguanidine (MIBG) imaging. Its central sympathetic is the most well-studied and efficient diagnostic technique that may be used to diagnose premotor PD (Okano et al., 2017; De Pablo-Fernández et al., 2017).

### 7.4 Fluorescent probe-based imaging markers

At present, the most common fluorescence probe targets for PD biomarkers include *α*-Syn, type B monoamine oxidase (MAO-B), dopamine-associated proteins (DATs and DARs), metal ions, ROS clusters, etc. However, these targets are just subjected to researches *in vitro* and at the animal level, without clinical applications. The existing deficiencies obstructing the use of fluorescent probes as diagnostic markers for PD are low biocompatibility, poor BBB permeability, unstable fluorescence properties, and poor receptor selectivity (Sun et al., 2024) (Table 6).

**Table 6:** Imaging biomarkers in patients with PD.

Imaging biomarkers	Compared to healthy subjects	Diagnostic value	References
Ultrasound technology	High echo black quality	84% sensitivity and 85% specificity	Tao et al. (2019)
NM-MRI	NM signal volume reduction	Sensitivity was 89% (95% CI 86%) −92%, with a mean specificity of 83% (95% CI 76 to 88%)	Mueller et al. (2014), Pyatigorskaya et al. (2018), Wang et al. (2018), Fabbri et al. (2017), Cho et al. (2020)
Free-water imaging	↑	Correlates with disease progression	Zhou et al. (2021), Ofori et al. (2015), Burciu et al. (2017)
DAT Production	↓	Sensitivity and specificity are 99–100%.	Palermo and Ceravolo (2019)
AADC imaging	↓	Associated with disease progression	Dhawan et al. (2022), Kaasinen et al. (2019)
VMAT2 imaging	↓	Correlates with severity of illness	Hsiao et al. (2014)
GLUT imaging	relative ↑ in metabolism in the pallidum-thalamus, pontine and cerebellum. Metabolism in premotor and posterior parietal regions ↓	93.9% sensitivity and specificity	Meles et al. (2019)
TSPO imaging	Nigrostriatal, pontine, basal ganglia, frontal and temporal cortex signaling ↑	Cannot be used to specifically diagnose early PD	Viviano et al. (2022), Alam et al. (2017)
SERT imaging	Binding of striatum, brainstem, and cortical regions ↓	Associated with pre-exercise pathology	Fu et al. (2022), Wilson et al. (2019)
^123^I-MIBG	↓	Related to activities of daily living (ADLs)	Okano et al. (2017), De Pablo-Fernández et al. (2017)
Fluorescent probe	/	Currently limited to *in vitro* and animal studies, not yet clinically applicable	Sun et al. (2024)

## 8 Pathological markers

Considering the involvement of peripheral organs in PD, the colon, skin, gastrointestinal tract, and submandibular gland biopsies for *α*-Syn detection may all be appropriate substitutes for α-Syn.

### 8.1 Skin samples

Skin α-Syn is not able to distinguish between PD patients and healthy controls, which, however, may be highly efficient in identifying α-Syn seed activity in skin samples. In a study of α-Syn seed activity measurement in skin biopsies using RT-QuIC, detection of samples from the legs and back of the neck via skin bipsy had a sensitivity of 100% and specificity of 95% for the diagnosis of PD. Meanwhile, use of immunohistochemistry and immunofluorescence also achieved successful distinguishing of PD patients from healthy controls. Zamboni fixation, frozen sections, and immunofluorescence exhibited a sensitivity of 90% and specificity of >90% for p-α-Syn detection (Gibbons et al., 2016; Donadio et al., 2021). The emergence of Syn-One contributes to improved identification of α-Syn in skin samples, which is more closer to the clinical detection. This technique collects samples using a 3 mm impact biopsy tool to implement diagnosis using immunostaining for PGP 9.5 and p-α-Syn (pSer129) after Zamboni fixation, frozen sections, and immunofluorescence (Gibbons et al., 2022).

### 8.2 Salivary glands

According to a research based on submandibular gland needle core biopsy, α-Syn was present in 75% of PD cases. However, The positive rate of biopsy also reached approximately 30% of healthy controls. As a result, this indicator may not suitable for diagnosis separately; rather, it may be possible to use in conjunction with other tests (Adler et al., 2014).

### 8.3 Olfactory mucosa

There exist abundant α-Syn in the mucosa of the nose. Its sensitivity and specificity for diagnosing PD reached 95 and 91%, respectively. But great discrepancies have been reported in different studies (Beach et al., 2009; Witt et al., 2009), which necessitate more clinical data to bolster these findings.

### 8.4 Gastrointestinal tract

Pathogenic aggregates, p-α-Syn, fibrillar α-Syn (f-α-Syn), or Lewy pathology have been found in the upper and lower gastrointestinal tracts of PD patients, with α-Syn positive rates declining from proximal to distal. Moreover, all PD patients had *α*-Syn positive submucosal biopsies of the colon, and 80% of submucosal biopsies of the ascending colon showed positive staining for p-α-Syn in these patients. In contrast, 55% of PD patients had Lewy microsomes in the mucosa and submucosa, with the detection of Lewy fibers in submucosal biopsies of the rectum as well as the ascending and descending colon in 23–65% of PD patients. Therefore, the rectum and sigmoid colon may not be suitable specimens for early diagnosis of PD. The diagnostic specificity of PD may be enhanced by p-*α*-Syn, o-α-Syn, f-α-Syn, o-α-Syn/t-α-Syn, or p-α-Syn/t-α-Syn, and tissue biopsies obtained during gastroenteroscopy may serve as possible markers for PD diagnosis (Lebouvier et al., 2010; Pouclet et al., 2012b; Pouclet et al., 2012a).

### 8.5 Retinass

Retinal non-myelinated axons are directly related to the CNS, offering an important method for studying neurological diseases. Pathological alterations in the retinal nerve fiber layer (RNFL) exhibit an intimate association with neurological disorders. Optical coherence tomography (OCT) facilitates the measurement of tissues at micrometer resolution. Patients with PD had thinner average RNFLs, indicating potential diagnostic significance of RNFL thickness (Alves et al., 2023) (see Table 7).

**Table 7:** Biomarkers of pathology in patients with PD.

Pathology biomarkers	Compared to healthy subjects	Diagnostic value	References
Skin alpha-synuclein	↑	>90% sensitivity and > 90% specificity	Gibbons et al. (2016), Donadio et al. (2021)
Alpha-synuclein accumulation in submandibular gland	↑	Higher false positive rate, cannot be used as a diagnostic biomarker alone, combined with other tests	Adler et al. (2014)
Olfactory bulb alpha-synuclein	↑	95% sensitivity, 91% specificity	Beach et al. (2009)
Lewy’s pathology of the colon and rectum intestines	+	Rectal biopsies are significantly less sensitive than ascending colon biopsies for detecting intestinal Lewy’s pathology.	Lebouvier et al. (2010), Pouclet et al. (2012b), Pouclet et al. (2012a)
Retinal nerve fiber layer (RNFL)	Thickness ↓	High value in early appraisals	Alves et al. (2023)

#### 8.5.1 Integration of different biomarkers to benefit early diagnosis of PD

The performance of combined biomarkers can be further enhanced by integrating demographic and clinical characteristics. Given an inferior performance of individual biomarkers in diagnosing early PD, combination of multiple biomarkers may greatly improve the diagnostic efficacy. Multiple studies have been conducted to decipher the combination of multiple humoral biomarkers. However, it is uncommon and challenging to statistically assess the combination of clinical, imaging, biochemical, pathological, genetic, and electrophysiological markers. In the future, more large-scale clinical trials are required on the basis of collaboration with mathematics, computer, and other specialties. Additionally, in order to identify the most effective diagnostic combinations and investigate their potential clinical significance, there is a need to employ the currently popular machine learning techniques to aggregate biomarkers from various sources. To achieve early diagnosis and overall management of PD, key techniques and challenges include constructing a clinical database, bolstering PD early biomarker research, interpreting the clinical significance of biomarkers, enhancing interdisciplinary collaboration, establishing a joint diagnosis and AI diagnostic system using multiple biomarkers, as well as setting up a one-stop diagnostic and treatment center in partnership with community-based health service centers to screen, manage, and educate the high-risk PD populations.

A combination of assays, including CSF *α*-syn and other biochemical indicators, MRI imaging markers, SPECT or PET imaging, non-motor symptoms, and cognitive function scores, should be utilized to detect early PD because individual biomarkers are difficult to diagnose reliably. In the latter stages of the disease, these tests can distinguish PD from other neurologic disorders and show how the disease is progressing. They are also important in the early stages of PD. Thus, future research could look at a complete system of precise, affordable, and non-invasive biomarkers for early PD.
